# Supplementary material for: Spheroids Composed of Reaggregated Neonatal Porcine Islets and Human Endothelial Cells Accelerate Development of Normoglycemia in Diabetic Mice
Source: Cells. 2025 Mar 2;14(5):366. doi: 10.3390/cells14050366 (PMC11898817; doi:10.3390/cells14050366)
Supplement: Supplementary file 1 [file cells-14-00366-s001.zip › cells-3155738-supplementary.pdf]

Supplemental Table S1. Primer sequences for quantitative real time-PCR.

Gene symbol	Primer sequence (5′-3′)	Amplicon length (bp)	Source
TNFAIP3 (A20)	F: AAGTGAGGAAGCTTGTGGCA<br>R: ATTCCTCAGTCCAATTCCGGG	240	XM_021077624.1
HMOX1	F: CGCTCCCGAATGAACACTCT<br>R: TTGCCACCAGAAAGCTGAGT	292	NM_001004027.1
B2M	F: CAAGATAGTTAAGTGGGATCG<br>R: TGGTAACATCAATACGATTTC	161	NM_213978.1
VEGFA	F: ATGCGGATCAAACCTCACCA<br>R: CACCAACGTACACGCTCCAG	187	NM_001025366.3
TM7SF2	F: GGTCAATGGCTTCCAGTTGCTC<br>R: AACGCCAGCATGAAGCCAAACC	108	NM_003273.6
GAPDH	F: CCACATCGCTCAGACACCAT<br>R: GCAACAATATCCACTTTACCAGAGT	114	NM_002046.7

TNFAIP3: porcine TNF alpha induced protein 3 (A20); *HMOX1*: porcine heme oxygenase (HO)-1; *B2M:* porcine beta-2-microglobulin;

VEGFA: human vascular endothelial cell growth factor; *ANG1:* human angiopoetin-1; *GAPDH:* human glyceraldehyde 3-phosphate dehydrogenase;

Supplemental Figure S1

A

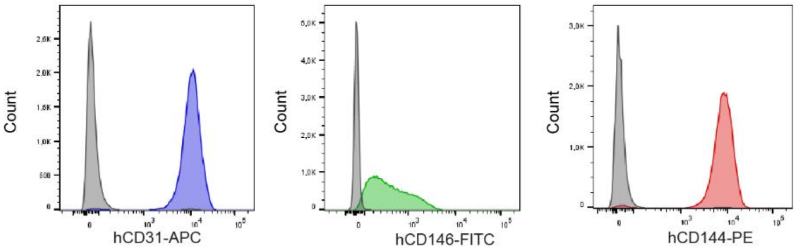

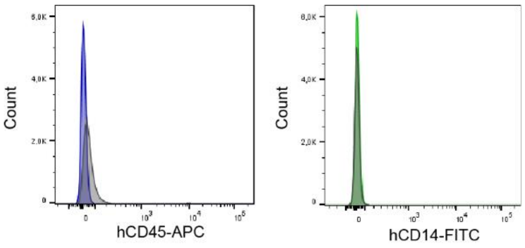

B

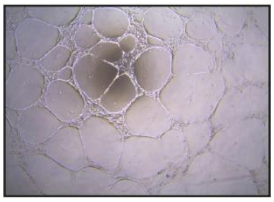

Supplemental Figure S1. Phenotype of isolated cord blood derived blood outgrowth endothelial cells (BOECs).

(**A**) BOECs expressed markers of endothelial cells (CD31, CD144, CD146), but not leukocyte (CD45) or monocyte (CD14) markers as assessed by flow cytometry. (**B**) Seeding of BOECs on Matrigel results in the formation of tube-like structures.

Supplemental Figure S2

A

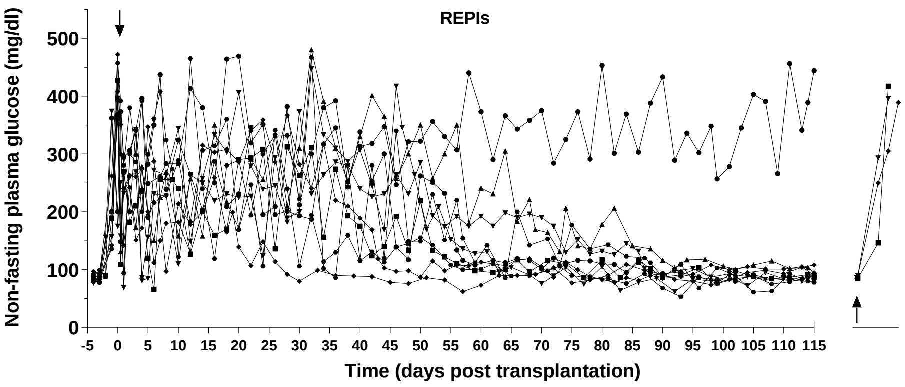

B

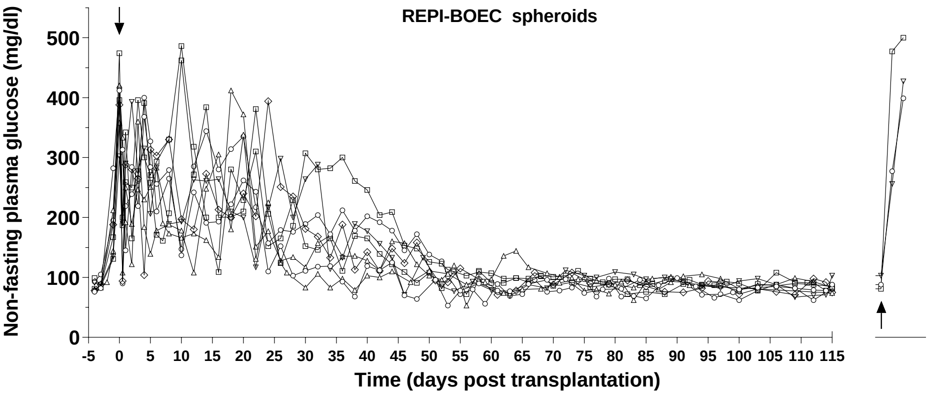

C

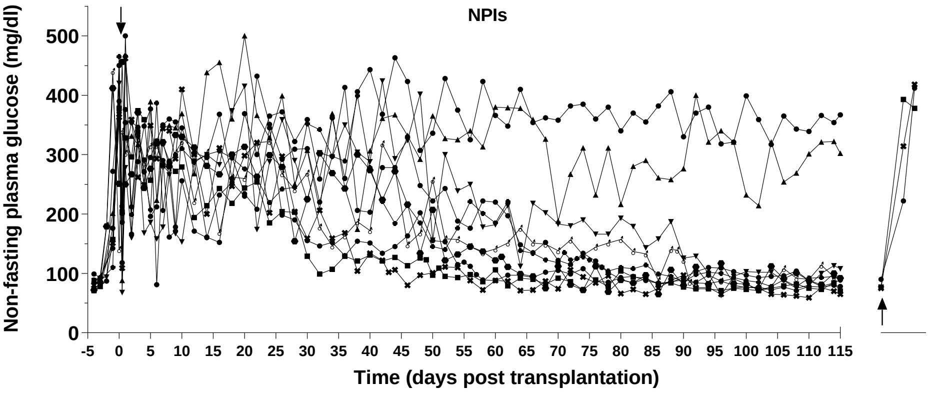

Supplemental Figure S2. Non-Fasting blood glucose measurements of diabetic NSG mice after transplantation.

(**A**) Transplantation of 1500 reaggregated neonatal porcine islet cells (REPIs). (**B**) Transplantation of 1500 REPI-BOEC spheroids. (**C**) Blood glucose profiles after transplantation of 3000 neonatal porcine islets (NPIs) are given for comparison. Arrows indicate day of transplantation and day of nephrectomy of the transplant bearing kidney (n= 3 animals in each transplantation group).
